# Angiosarcoma in the Setting of Vascular Procedures: A Systematic Review and Our Experiences

**DOI:** 10.1093/icvts/ivag236

**Published:** 2026-08-24

**Authors:** Phil Yi Jun Lu, Joel Ding, Casey Hiu Ching Fung, Michelle Davis, Mark Westcott, Justin Jedynak, Yahya Lahham

**Affiliations:** Vascular Surgery, Department of Surgery, Alfred Health, Melbourne, VIC 3004, Australia; Vascular Surgery, Department of Surgery, Frankston Hospital, Peninsula Health, Melbourne, VIC 3199, Australia; Vascular Surgery, Department of Surgery, St Vincent’s Hospital, Melbourne, VIC 3065, Australia; Cardiothoracic Surgery, Department of Surgery, Austin Health, Melbourne, VIC 3084, Australia; Vascular Surgery, Department of Surgery, Frankston Hospital, Peninsula Health, Melbourne, VIC 3199, Australia; Vascular Surgery, Department of Surgery, St Vincent’s Hospital, Melbourne, VIC 3065, Australia; Melbourne Medical School, University of Melbourne, Melbourne, VIC 3010, Australia; Vascular Surgery, Department of Surgery, Frankston Hospital, Peninsula Health, Melbourne, VIC 3199, Australia; Vascular Surgery, Department of Surgery, Alfred Health, Melbourne, VIC 3004, Australia; Vascular Surgery, Department of Surgery, Frankston Hospital, Peninsula Health, Melbourne, VIC 3199, Australia; Vascular Surgery, Department of Surgery, St Vincent’s Hospital, Melbourne, VIC 3065, Australia

**Keywords:** angiosarcoma, vascular reconstruction, endovascular aneurysm repair, vascular prostheses, bypass graft, systematic review

## Abstract

**Objectives:**

Angiosarcoma is a rare, aggressive endothelial malignancy with a poor prognosis; diagnosis is often delayed by vague symptoms, nonspecific imaging, and challenging histopathology. Case reports have suggested an association with prior vascular procedures, with or without prosthetic material, but no causal link is established. We reviewed angiosarcoma across the full spectrum of vascular reconstruction.

**Methods:**

We systematically reviewed English-language human studies in PubMed, Embase, and the Cochrane Library for histologically confirmed angiosarcomas diagnosed after and anatomically related to vascular procedures. The review was registered on PROSPERO (CRD42024602643).

**Results:**

We describe 3 patients with metastatic angiosarcoma contiguous with vascular prostheses: a 75-year-old male with left inguinal angiosarcoma 7 years after PTFE bypass grafting; a 69-year-old male with angiosarcoma within the aneurysm sac 7 years after endovascular aortic aneurysm repair (EVAR); and an 80-year-old male with intraluminal angiosarcoma within an EVAR limb presenting as distal embolization. Systematic review identified 44 studies comprising 46 additional cases. Including these, 49 patients were analysed: 27 associated with endovascular prostheses, 14 with open prosthetic or mixed grafts, and 8 with autologous-vein bypass. Mean diagnostic latency was 7 years (range 5 months to 18 years); mean age was 70.5 years; 12.2% were female.

**Conclusions:**

Angiosarcoma is difficult to diagnose given its nonspecific presentation but should be considered in atypical or refractory post-procedural presentations. This is the largest synthesis to date and the first to describe intraluminal EVAR-limb angiosarcoma with distal embolization. Inclusion of autologous-vein reconstructions argues against a purely prosthetic-material explanation; further investigation is needed to clarify causality.

## Graphical abstract

**Figure:**
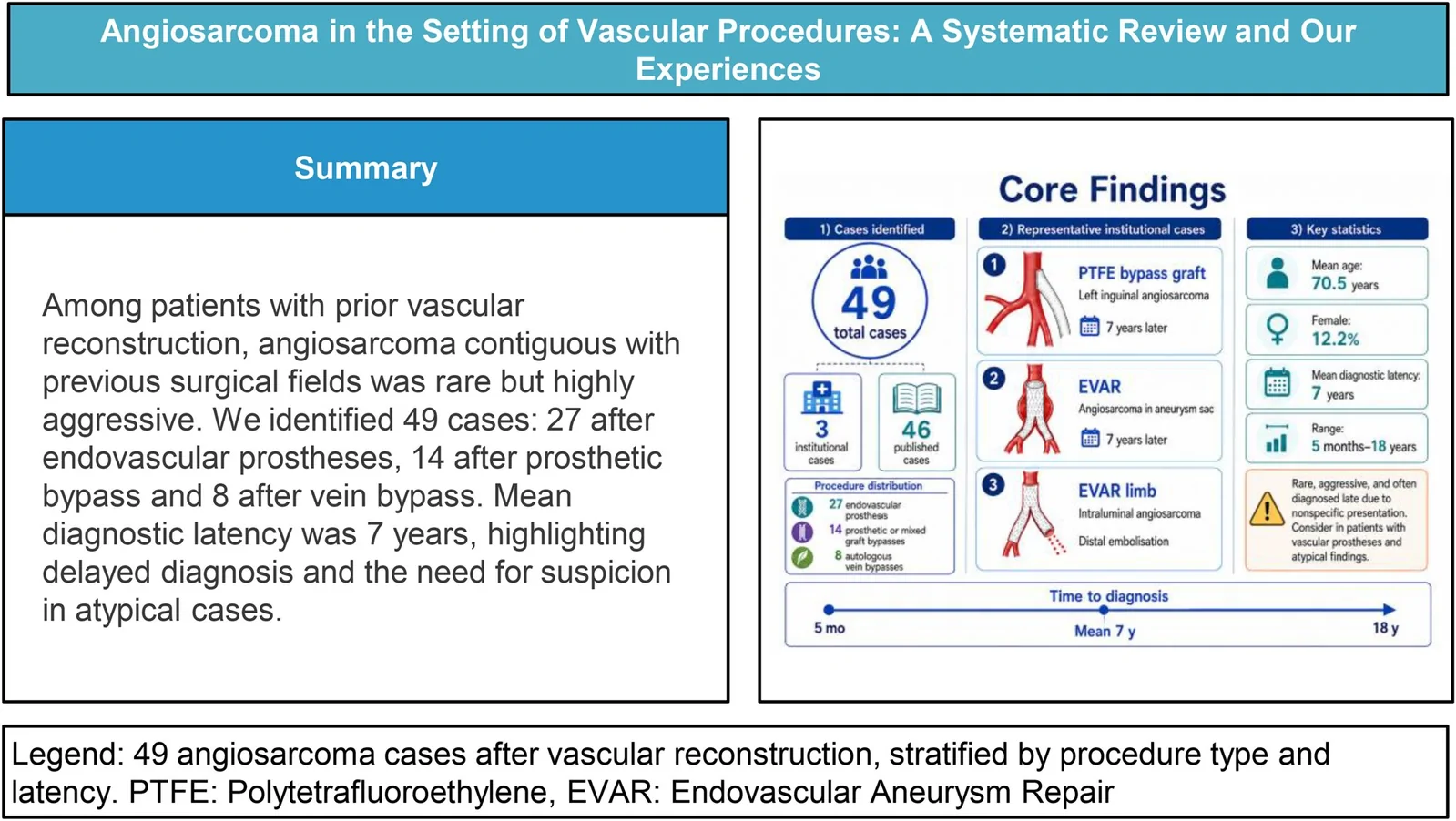

## INTRODUCTION

Primary aortic malignancies including angiosarcomas are a rare but aggressive form of tumour that are often metastatic at the time of diagnosis. Angiosarcomas are thought to comprise less than 2% of soft tissue sarcomas with an overall incidence of less than 0.15 per 100 000 person years,^1^ with under 200 primary aortic angiosarcomas reported in the literature.^2^^,^^3^ Patients often present with non-specific symptoms including abdominal or lower back pain and weight loss. Imaging is also often nondiagnostic and may mimic more common conditions such as endovascular aortic aneurysm repair (EVAR) endoleak or graft infection. The diagnosis of angiosarcoma is therefore challenging and relies heavily on histopathological examination. Diagnosis is usually only made post metastatic spread or post-mortem. Current treatment options are limited and have a poor prognosis; it is thought that radical surgical resection with adjuvant chemotherapy carries the best prognosis when possible.^3^^,^^4^ Five-year survival rates are estimated at 30%.^5^^,^^6^

The pathophysiology of angiosarcoma remains poorly understood. However, established risk factors such as radiation exposure, chronic lymphoedema, environmental carcinogens, and genetic syndromes are thought to contribute to its development.^7^^,^^8^ These factors may contribute to angiosarcoma development through chronic inflammation, a mechanism hypothesized to be involved in angiosarcoma pathogenesis and frequently observed histopathologically.^9^ Additionally, it has been hypothesized that foreign material within the intima results in turbulent flow with chronic injury, thereby promoting an environment of rapid cell turnover and oncogene activation.^10^ Nevertheless, primary angiosarcomas have also been reported to arise de novo without recognized risk factors, suggesting that spontaneous tumourigenesis, although exceedingly rare, may also occur.^11–13^

An increasing number of case reports on angiosarcomas arising in close physical proximity to implanted prostheses, in both open vascular and endovascular procedures,^14^ have been published in the literature. Synthetic polymers including Dacron have been implicated as carcinogenic and capable of inducing sarcoma in animal studies,^15^ but currently a causative link has not yet been established in humans.^14^

This systematic review aims to evaluate the existing literature on angiosarcoma arising after vascular procedures across the full spectrum of vascular reconstruction, including endovascular prostheses, open prosthetic grafts, and autologous-vein conduits. Specifically, we reviewed the patient demographics, type and duration of prosthetic implantation (if any), angiosarcoma pathology, treatment approaches, and clinical outcomes of patients with diagnosed angiosarcoma following vascular procedures.

In addition to the systematic analysis of the literature, we provide a case series of 3 patients who were diagnosed with primary angiosarcoma contiguous with previous vascular prostheses. Two patients had metastatic disease at diagnosis, and all 3 died within months of diagnosis or recurrence.

The novelty of this manuscript is 3-fold. First, we describe an apparent intraluminal angiosarcoma within an EVAR limb presenting with distal embolization, a presentation not identified among the other included reports and one that expands the recognized clinical spectrum of this rare entity. Second, to our knowledge, this is the largest synthesis specifically focused on angiosarcoma reported after vascular procedures. Third, by deliberately including endovascular prostheses, open bypasses with prosthetic or autologous-vein conduits, this review allows the reported association to be examined across the full vascular reconstruction spectrum. The inclusion of autologous-vein cases is particularly important because it challenges a purely prosthetic-material hypothesis and supports a broader, hypothesis-generating discussion of local tissue injury, chronic inflammation, altered haemodynamics, cumulative radiation exposure, and spontaneous tumourigenesis.

## METHODS

Study identification, screening, eligibility assessment, and inclusion were performed in accordance with PRISMA 2020 guidance. The study-selection process is shown in **Figure 1**. A systematic literature search was performed across databases including PubMed, Embase, and the Cochrane Library from database inception to October 2024. The search strategy combined keywords and Medical Subject Headings (MeSH) terms such as “angiosarcoma,” “primary vascular tumours,” “vascular prosthesis,” “stent,” “EVAR,” “aorta,” “bypass graft,” “Dacron,” and “PTFE.” Boolean operators were used to refine the search, and language restrictions were set to include only English-language articles involving human subjects.

**Figure 1.**
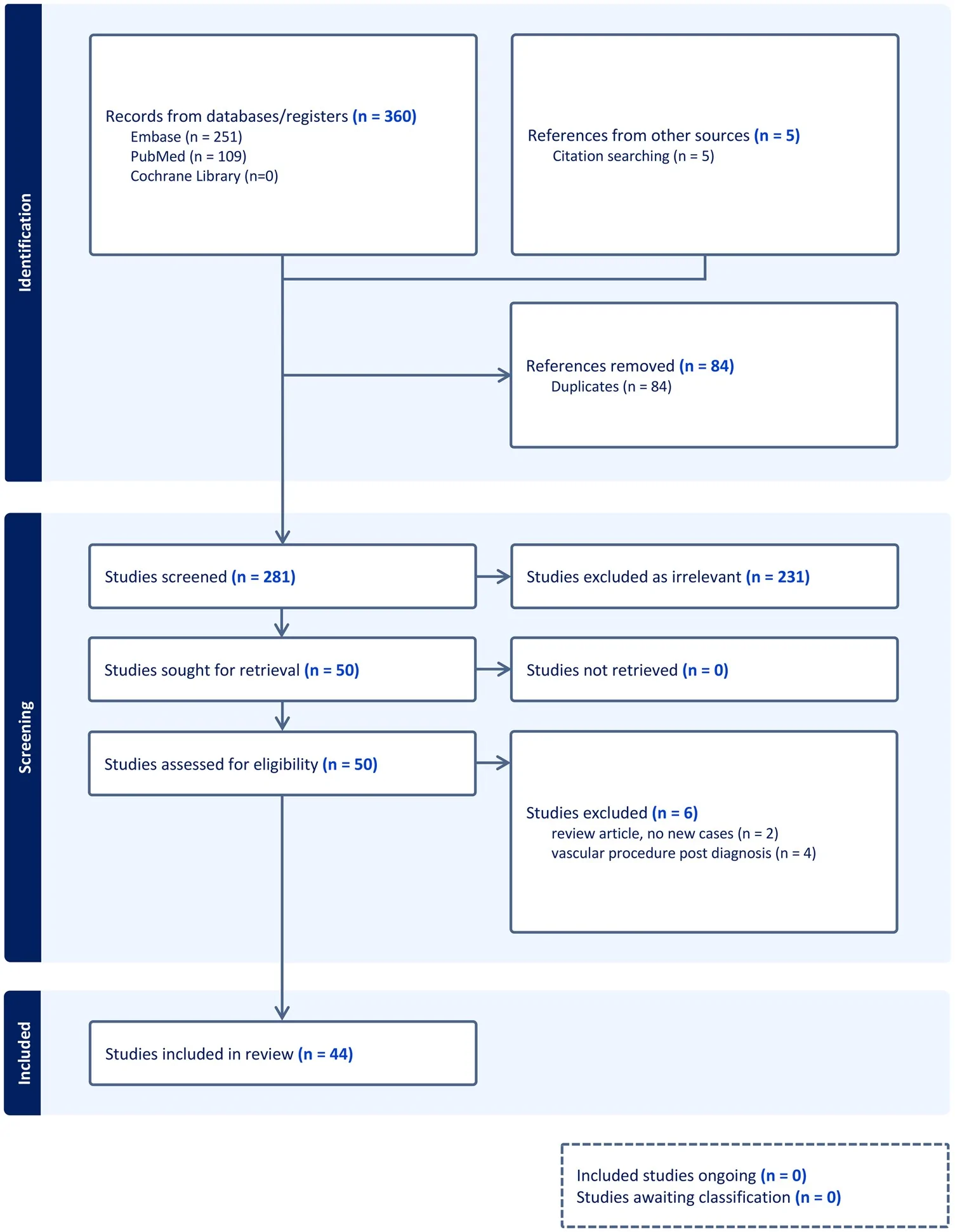
PRISMA 2020 Flow Diagram of Study Identification, Screening, Eligibility Assessment, and Inclusion. Database searching identified 360 records and citation searching identified 5 additional records. Following removal of 84 duplicates, 281 records underwent title and abstract screening. Fifty reports were assessed in full text, with 6 excluded for predefined reasons, leaving 44 published studies for inclusion in the systematic review

The search process is summarized in the PRISMA flow diagram (**Figure 1**). Records identified from database searching and citation searching were exported into EndNote for deduplication. Duplicate records were removed before title and abstract screening using automated duplicate detection followed by manual verification of author names, title, journal, year, and DOI where available. Two reviewers independently screened titles and abstracts, then assessed potentially eligible full-text reports. Disagreements were resolved by discussion and consensus. Reasons for exclusion at full-text review were recorded prospectively and are presented in **Figure 1**. The complete reproducible search strategy for each database is provided in **Supplementary File 1**.

Studies were eligible if they described cases of histologically confirmed angiosarcoma that were (1) diagnosed after a vascular procedure; (2) anatomically related to the treated vessel, graft, or conduit; (3) occurred after an endovascular prosthesis or reconstruction with a prosthesis or autologous vein. Exclusion criteria included unrelated cases of angiosarcoma (not following vascular procedures, or diagnosed prior to or during the index operation), duplicate studies, and studies on animal models.

All references from selected articles were analysed to identify further studies. Due to the rarity of angiosarcoma and the expected heterogeneity of case reports and series only, findings were synthesized in a narrative format.

Data extraction focused on variables such as patient demographics, symptoms, prosthesis type (eg, PTFE, Dacron, endovascular), anatomical site, latency from procedure to tumour detection, metastatic status at diagnosis, and treatment modalities.

To complement the systematic review, a case series of 3 patients is presented, detailing clinical characteristics, prosthetic history, histopathology, treatment strategies, and outcomes. Data were retrospectively gathered from medical records to provide context-specific insights into angiosarcomas reported after vascular procedures. Informed consent from patients and their next of kin was obtained for the publication of this anonymized case report, including the use of clinical data and accompanying images.

This study comprises a systematic review of the published literature and a retrospective case series of 3 patients. In accordance with institutional policy and national guidelines, formal Human Research Ethics Committee approval was not required for the systematic review component, as it involved analysis of previously published, de-identified data. The case series component was conducted in accordance with the ethical principles set forth in the Declaration of Helsinki. Written informed consent for publication was obtained from the next of kin of each patient included in the case series.

### Assessment of reporting completeness

Given that all included studies were case reports or small case series, a structured assessment of reporting completeness was performed rather than a conventional comparative risk-of-bias assessment. Each included case was assessed using predefined domains adapted from case-report appraisal frameworks, including patient demographics, clinical history, index vascular procedure, conduit or prosthesis type, anatomical relationship between the tumour and prior vascular reconstruction, time from procedure to tumour detection, diagnostic modality, histopathological and immunohistochemical confirmation, treatment, outcome, and duration of follow-up. Missing or insufficiently reported data were recorded as not stated. Reporting completeness was used to contextualize the certainty of the descriptive synthesis and was not used to exclude studies.

### Statistical analysis

Given the case-report design, small sample size, heterogeneity, and incomplete reporting, no meta-analysis or inferential statistical testing was performed. Data were summarized descriptively. Categorical variables are reported as counts and percentages, and continuous variables as mean, median and range where available. Missing or insufficiently reported data were recorded as not stated and excluded from denominator-specific calculations where appropriate.

### Case series

#### Patient 1

A 75-year-old Caucasian male presented emergently with hypotension and a 2-month history of intermittent suprapubic abdominal and midline back pain, recurrent syncopal episodes, 15 kg of unintentional weight loss, and nausea. He had a complex vascular surgical history including an open left femoral artery aneurysm repair with an interposition ring-reinforced PTFE graft (Propaten, W. L. Gore & Associates) at age 62, a stent to the left femoropopliteal segment for an aneurysm at age 66 (Viabahn, W. L. Gore & Associates), a left common iliac aneurysm treated with EVAR and an iliac branched device (Zenith Alpha, Cook Medical) at age 67, and a right femoral to left below knee popliteal PTFE bypass graft (Propaten) at age 68 for a thrombosed left common femoral artery at the site of previous open repair. His other medical conditions included hypertension, hypercholesterolaemia, and ischaemic heart disease.

His blood tests were unremarkable, with non-elevated tumour markers. On computed tomography (CT) imaging, a potential metastatic lesion in the left acetabulum was identified. A subsequent 18 F-fluorodeoxyglucose positron emission tomography (18 F-FDG PET) scan demonstrated a high avidity lesion adjacent to the PTFE graft in the left inguinal region suggestive of primary malignancy, with evidence of distal metastases in the pelvis, vertebral bodies, and lower limbs. A CT-guided core biopsy of a sacral ala lesion suspected to be a metastasis was performed for histopathological examination. Microscopy showed atypical epithelioid cells with slit-like spaces and stromal infiltration. Immunohistochemistry demonstrated positive staining for AE1/3, CD31, and factor VIII, while CD34 and melanocytic markers (S100, SOX10) were negative consistent with epithelioid angiosarcoma.

Due to significant comorbidities and extensive metastatic disease, surgical intervention was deemed unsuitable, and he was commenced on palliative chemotherapy. The patient passed away 2 months after diagnosis.

#### Patient 2

A 69-year-old male with a history of elective EVAR (Zenith Flex, Cook Medical) for a 60 mm infrarenal abdominal aortic aneurysm (AAA), presented for routine follow-up 7 years post-implantation. He reported non-specific abdominal pain and loss of appetite. Computed tomography angiography revealed aneurysm sac expansion without obvious endoleak. Digital subtraction angiography (DSA) also did not demonstrate an obvious endoleak, but prophylactic inferior mesenteric artery embolization was performed nonetheless given ongoing sac expansion. Subsequent CT-guided direct sac puncture and Histoacryl glue embolization was also performed.

The patient re-presented 6 months later with worsening abdominal pain, anorexia, and a 40 kg weight loss. Laboratory studies showed a normal leucocyte count, with mildly elevated C-reactive protein (66 mg/L). Tumour markers were negative. A repeat CT abdominal aorta angiogram indicated subtle sac growth (67 mm to 69 mm) with new para-aortic fat stranding without signs of rupture. Subsequent nuclear medicine scans and FDG-PET scan demonstrated intense uptake around the aortic endoprosthesis and L3 vertebra with radiological features of graft infection and osteomyelitis. Two initial CT-guided biopsies of the L3 vertebra were initially non-diagnostic due to tissue fragmentation and sampling limitations, with reports of few atypical epithelioid cells and no microbiological growth. A third percutaneous biopsy identified poorly differentiated epithelioid angiosarcoma, positive for cytokeratin AE1/AE3, CD31, and ERG on immunohistochemistry.

Chemotherapy was promptly initiated. There was initially a complete metabolic response; however, 12 months later, he was readmitted with dyspnoea secondary to recurrence with extensive pulmonary metastases and microhaemorrhages. He died shortly thereafter.

#### Patient 3

An 80-year-old male with a previous infrarenal EVAR (Zenith Flex, Cook Medical) performed 15 years prior presented with left hallux tissue loss and new stenoses within bilateral iliac limbs of his endovascular stent graft. Relevant past medical history included coronary artery bypass grafting and atrial flutter on anticoagulation. Of note, no type 1 or type 3 endoleaks had been noted on serial surveillance imaging.

Balloon angioplasty of the iliac limbs was thus performed, but this was complicated by distal embolization of the left leg requiring emergent popliteal and tibial embolectomy. Atypical pale gelatinous debris was retrieved from the posterior tibial artery, which was later found to be epithelioid angiosarcoma on histopathology, positive for CD31 and ERG on immunohistochemistry.

Subsequent 18 F-FDG PET scan demonstrated heterogeneous activity within the lumen of the in-situ EVAR suggestive of primary aortic angiosarcoma (**Figure 2A–C**). Metastatic disease was also suspected involving the L4 vertebral body and bilateral thigh muscles. Given the patient’s age, comorbidities, and the presence of metastases, palliative radiotherapy and chemotherapy with paclitaxel was pursued. The patient subsequently developed haemoptysis while undergoing chemoradiotherapy, and further CT scans revealed new bilateral pulmonary and cerebral metastases also, suggestive of highly aggressive disease. The patient was referred for palliative care and died 2 months after diagnosis of angiosarcoma.

**Figure 2.**
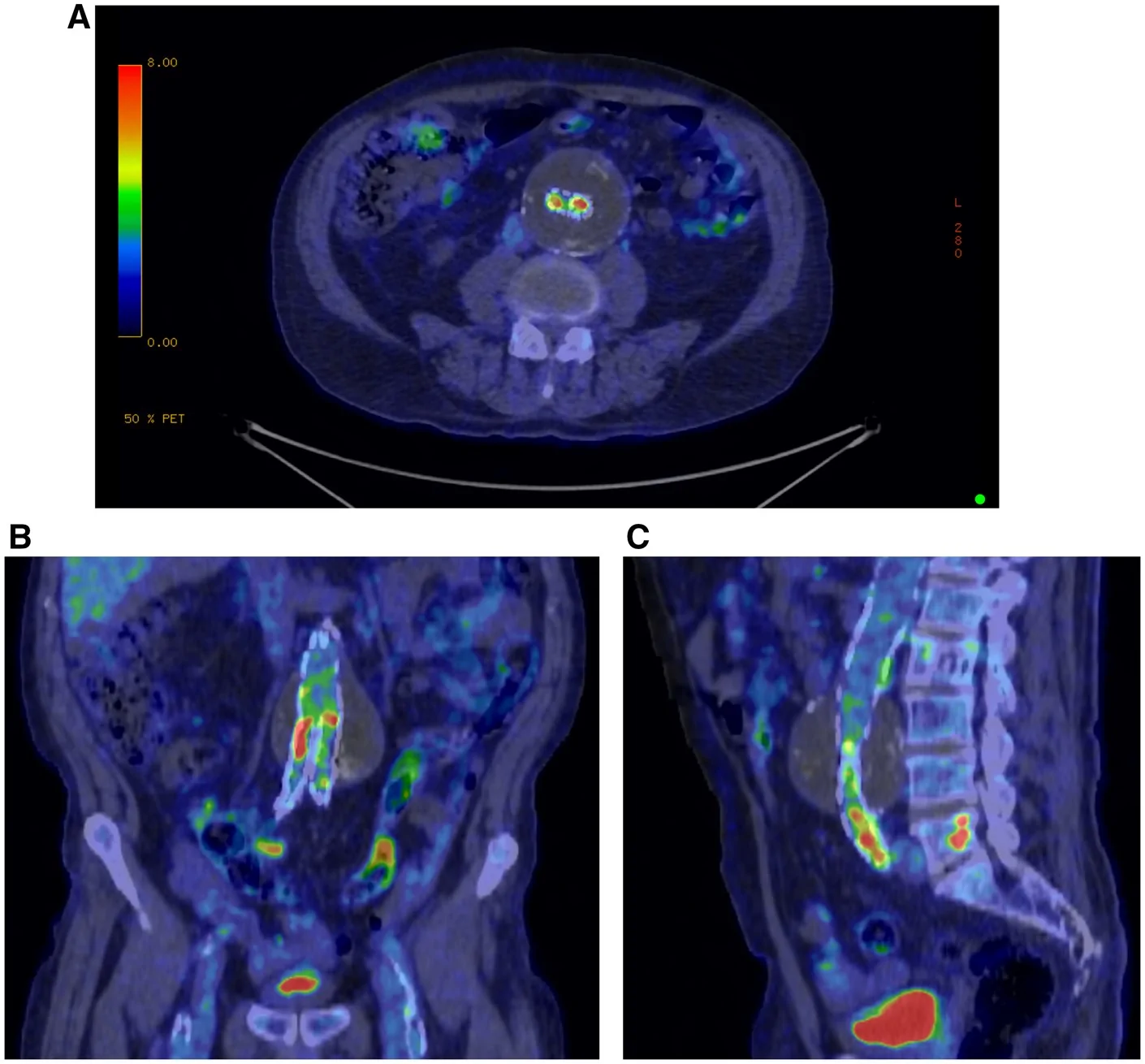
Axial (A), coronal (B), and sagittal (C) FDG-PET/CT views of the intraluminal angiosarcoma within the limbs of the existing EVAR graft in Patient 3. Note the avidity is confined within the lumen of the stent graft itself, with no avidity seen in the aortic sac

### Results of systematic review

The search identified 360 records from database searching and 5 additional records through citation searching. After removal of 84 duplicates, 281 records underwent title and abstract screening. Of these, 231 were excluded as irrelevant. Fifty full-text reports were assessed for eligibility, of which 6 were excluded: 2 were review articles with no additional cases and 4 described vascular procedures performed after the diagnosis of angiosarcoma. Forty-four published studies were included in the systematic review, representing 46 published patients. Together with the 3 institutional cases, 49 patients were included in the final descriptive synthesis.

A total of 44 studies were identified in the systematic search and were included in this review. These studies represented a cohort of patients with confirmed angiosarcoma arising post-vascular surgery. All publications were case reports (all single reports with the exception of 2 articles which describe 2 cases each). Reporting completeness varied substantially across included reports. Age, sex, and presenting symptoms were usually reported, whereas conduit material, exact anatomical relationship to the previous vascular reconstruction, time from index procedure to tumour detection, metastatic status, and follow-up duration were inconsistently described. Histological confirmation was reported in all included cases, although the extent of immunohistochemical characterization varied between reports. These limitations informed the decision to present the findings descriptively and to avoid causal or incidence-based conclusions.

In total, 49 patients were identified including the 3 identified in this case report. Twenty-seven were related to endovascular prostheses (primarily aortic procedures) as outlined on **Table 1**, 14 were related to open bypasses using prosthetic grafts as outlined on **Table 2**, and 8 related to open arterial bypasses using autologous vein as outlined on **Table 3**. Patients were mostly male (42 males, 6 females, and 1 not stated). The mean age of diagnosis was 70.5 years (range 50 to 86 years). Time from vascular procedure to tumour detection was reported in 46 cases, with a mean of 7.1 years, median of 7.0 years, and range of 5 months to 18 years.

**Table 1.** Review of Literature: Angiosarcoma Following Endovascular Procedure

	Age	Sex	Type of operation	Type of material	Prosthesis	Symptoms	Imaging	Differential diagnoses	Diagnosis made by	Time to tumour detection	Site of primary	Metastasis at time of diagnosis	Treatment	Outcome	Time to outcome
Almajan et al (2021)^16^	69	F	Endovascular	Polyester	Endurant Endograft (Medtronic)	Back pain	CT/CT A; DSA	Endoleak	Surgical explant	3 years	Abdominal aorta	Yes	Surgery; chemotherapy	Deceased	3 months
Kano et al (2019)^17^	82	M	Endovascular	NS	Self-expanding stents (not stated)	Lower limb claudication	CT/CT A	CTLI	Surgical explant	7 months	Iliac	Yes	Surgery; chemotherapy; radiotherapy	Deceased	20 months
Schmehl et al (2012)^18^	77	?	Endovascular	Polyester	Talent Endoprosthesis (Medtronic)	Abdominal pain; weight loss; fatigue	PET-FDG	Endoleak	Surgical explant	7 years	Abdominal aorta	Yes	Surgery; chemotherapy	Unknown	NS
Fatima et al (2013)^12^	57	M	Endovascular	NS	Prosthetic bifurcated aortobifemoral bypass graft (not stated)	Back pain; weight loss; fever; chills; night sweats	CT/CT A	Psoas abscess	Surgical explant	6 years	Abdominal aorta	Yes	Surgery	Deceased	2 months
Umscheid et al (2007)^19^	50	M	Endovascular	Polyester	Vanguard Bifurcated Stent-Graft (Boston Scientific)	Abdominal pain; palpable mass; weight loss; PR bleeding/melaena	CT/CT A; Endoscopy	Graft infection; fistula; contained rupture	Surgical explant	5 years	Abdominal aorta	Yes	Surgery; chemotherapy	Deceased	24 months
Stewart et al (2013)^20^	86	M	Endovascular	Polyester	Ancure Endovascular Bifurcation Unibody Aortic Graft (Guidant Corporation)	PR bleeding/melaena	CT/CT A; Colonoscopy	Endoleak; fistula	Surgical explant	9 years	Abdominal aorta	Yes	Surgery	Deceased	3 months
Fenton et al (2014)^21^	66	M	Endovascular	Polyester	AneuRx Endovascular Prosthesis (Medtronic)	Back pain; weight loss	CT/CT A	Graft infection	Percutaneous biopsy	6 years	Iliac	Yes	Chemotherapy; radiotherapy	Deceased	1 month
Kimura et al (2015)^22^	78	M	Endovascular	Polyester	Prosthetic aortic graft (woven Dacron)	Fever	CT/CT A	Graft infection; pseudoaneurysm	Surgical explant	16 years	Thoracic aorta	No	Surgery	Deceased	2 months
Milite et al (2016)^23^	60	M	Endovascular	PTFE	Excluder Endoprosthesis (W. L. Gore & Associates)	Abdominal pain; back pain; fever; fatigue	CT/CT A	Graft infection	Surgical explant	7 years	Abdominal aorta	Yes	Surgery	Deceased	1 months
Tiwari et al (2016)^24^	64	M	Endovascular	Polyester	NS	Weight loss; fever; dyspnoea; painful skin eruption	MRI/MRA; TOE, gastroscopy, endoscopy, White cell nuclear medicine scan	Endoleak	Percutaneous biopsy	7 years	Abdominal aorta	No	Chemotherapy	Unknown	3 months
Kamran et al (1) (2016)^25^	69	M	Endovascular	NS	NS	Abdominal pain; weight loss; anorexia	CT/CT A; MRI/MRA; Gastroscopy, Colonoscopy, Capsule Endoscopy	Endoleak	Surgical explant	8 years	Abdominal aorta	No	Chemotherapy; radiotherapy	Alive	9 months
Kamran et al (2) (2016)^25^	72	F	Endovascular	NS	NS	Back pain	CT/CT A; White cell nuclear medicine scan (111In)	Endoleak; rupture	Percutaneous biopsy	7 months	Abdominal aorta	Yes	Chemotherapy	Unknown	NS
deAlmeida et al (2017)^26^	81	M	Endovascular	PTFE	Prosthetic endovascular bifurcated aortoiliac graft (not stated)	Abdominal pain; weight loss; dyspnoea; loss of appetite	CT/CT A	Endoleak	Surgical explant	5 years	Duodenum	Yes	Surgery	Deceased	2 days
Whittington et al (2019)^27^	78	M	Endovascular	PTFE	Excluder Endoprosthesis (W. L. Gore & Associates)	Abdominal pain; back pain; palpable mass	CT/CT A	Endoleak	Surgical explant	10 years	Abdominal aorta	Yes	None (palliative)	Deceased	Several months
Yu et al (2019)^28^	68	M	Endovascular	PTFE	Excluder Endoprosthesis (W. L. Gore & Associates)	Abdominal pain; back pain; fever	CT/CT A; PET-FDG	Graft infection; endoleak; arteritis; malignancy	NS	4 years	Abdominal aorta	Yes	Surgery; chemotherapy	Alive	2 months
Presacco et al (2020)^29^	84	M	Endovascular	PTFE	Ovation Abdominal Stent Graft System (TriVascular Inc.)	Back pain	CT/CT A; MRI/MRA	Graft infection; malignancy	Post-mortem	5 years	Abdominal aorta	No	Surgery	Deceased	2 months
Rota et al (2021)^30^	72	M	Endovascular	NS	Undisclosed bifurcated stent graft (W. L. Gore & Associates)	Back pain; fever	CT/CT A; PET-FDG	Graft infection; endoleak	Surgical explant	8 years	Abdominal aorta	No	None (palliative)	Deceased	1 months
Derouane et al (2021)^31^	68	M	Endovascular	Polyester	NS	Lower limb claudication	CT/CT A; US	Endoleak	Surgical explant	3.5 years	Iliac	No	Surgery; chemotherapy	Deceased	5 weeks
Takamura et al (2021)^32^	82	F	Endovascular	PTFE	TEVAR (not stated)	Fever	CT/CT A	Graft infection; endoleak	Post-mortem	4 years	NS	No	None (palliative)	Deceased	2 months
Walker et al (2021)^33^	69	M	Endovascular	NS	Bifurcating aorto-bi-iliac endoluminal stent graft	NS	CT/CT A; MRI/MRA; PET-FDG	Endoleak	Percutaneous biopsy	5 months	Abdominal aorta	Yes	Chemotherapy; radiotherapy	Alive	6 months
Pecceu et al (1) (2023)^34^	72	M	Endovascular	PTFE	Excluder Endoprosthesis (W. L. Gore & Associates)	Abdominal pain; back pain; weight loss	CT/CT A	Graft infection; endoleak	Surgical exploration	5 years	Iliac	No	Surgery; radiotherapy	Deceased	8 months
Pecceu et al (2) (2023)^34^	69	M	Endovascular	PTFE	Excluder Endoprosthesis (W. L. Gore & Associates)	Weight loss	CT/CT A; PET-FDG	Graft infection	Percutaneous biopsy	6 years	Abdominal aorta	Yes	None (palliative)	Deceased	2 weeks
Cleret de Langavant et al (2022)^35^	64	M	Endovascular	PTFE	AFX Bifurcated Endograft System (Endologix)	Back pain; leg pain	CT/CT A; PET-FDG; White cell nuclear medicine scan (99mTc)	Endoleak; psoitis; aneurysm progression; mural thrombosis	Surgical explant	4 years	Iliac	Yes	Surgery; chemotherapy	Deceased	Few weeks
Azuma et al (2024)^36^	66	M	Endovascular	PTFE	Excluder Endoprosthesis (W. L. Gore & Associates,)	Dyspnoea; hypotension	CT/CT A	Endoleak	Post-mortem	4 years	Abdominal aorta	Yes	Surgery	Deceased	17 days
Gegiia et al (2024)^37^	78	F	Endovascular	Polyester	Endurant II Endograft (Medtronic)	Abdominal pain; back pain; nausea; vomiting	CT/CT A	Contained aneurysmal sac rupture	Surgical explant	4 years	Abdominal aorta	Yes	Surgery	Deceased	4 months
This paper (2) 2025	69	M	Endovascular	Polyester	Zenith Flex Endovascular Graft (Cook Medical)	Abdominal pain; back pain; weight loss	CT/CT A; MRI/MRA; PET-FDG	Graft infection	Percutaneous biopsy	7 years	Abdominal aorta	No	Chemotherapy	Deceased	12 months
This paper (3) 2025	80	M	Endovascular	Polyester	Zenith Flex Endovascular Graft (Cook Medical)	Tissue loss	CT/CT A; PET-FDG	Graft thrombosis	Surgical thrombectomy	15 years	Abdominal aorta	Yes	Chemotherapy; Radiotherapy	Deceased	2 months

Abbreviations: CT: computed tomography; CT A: computed tomography angiography; MRA: magnetic resonance angiography; MRI: magnetic resonance imaging; NS: not stated; PET-FDG: positron-emission tomography—fluorodeoxyglucose; PTFE: polytetrafluoroethylene.

**Table 2.** Review of Literature: Angiosarcoma Following Open Procedure With Prosthetic Material

	Age	Sex	Type of operation	Type of material	Prosthesis	Symptoms	Imaging	Differential diagnoses	Diagnosis made by	Time to tumour detection	Site of primary	Metastasis at time of diagnosis	Treatment	Outcome	Time to outcome
Fehrenbacher et al (1981)^38^	67	M	Open	Polyester	Prosthetic bifurcated aortobifemoral bypass graft (Dacron)	Rest pain	DSA	Pseudoaneurysm; ALI	Surgical explant	12 years	Abdominal aorta	Yes	Surgery; chemotherapy	Deceased	7 months
Weiss et al (1991)^39^	56	M	Open	Polyester	Bifurcated woven double-velour Dacron graft (not stated)	Abdominal pain; back pain	CT/CT A; MRI/MRA	Graft infection; pseudoaneurysm	Surgical explant	3 years	Abdominal aorta	Yes	Surgery; chemotherapy	Alive	11 months
Brito et al (1996)^40^	77	M	Open	Polyester	Prosthetic bifurcated aortobifemoral bypass graft (Dacron)	Abdominal pain; back pain; weight loss; constipation	White cell nuclear medicine scan (99mTc)	Graft infection; pseudoaneurysm; malignancy	Percutaneous biopsy	NS	Iliac	No	None (palliative)	Deceased	NS
Dargent et al (1997)^41^	61	M	Open	Polyester	Prosthetic femorofemoral bypass graft (Dacron)	Arthralgias; myalgias; asthenia; gastrointestinal tract symptoms	CT/CT A	Malignancy	Post-mortem	NS	Iliac	Yes	Surgery	Deceased	9 days
Kogon et al (1998)^42^	70	M	Open	Vein, PTFE	Saphenous vein; prosthetic femoral to anterior tibial artery bypass graft (PTFE)	Palpable mass; fever; leg pain	MRI/MRA	Pseudoaneurysm	Surgical explant	13 years	Popliteal	No	Surgery; radiotherapy	Unknown	?
Ben-Izhak et al (1999)^43^	67	M	Open	Polyester	Aortobifemoral bypass graft (not stated)	Abdominal pain; palpable mass; weight loss; nausea; vomiting	CT/CT A	Malignancy	Percutaneous biopsy	8 years	Iliac	Yes	Surgery; chemotherapy	Deceased	6 months
Okada et al (2004)^44^	50	M	Open	Polyester	Dacron Aortic Root Graft (DeBakey Vasculour; C.R. Bard, Inc)	Stroke	CT/CT A	Malignancy	Surgical biopsy of metastatic lesion	17 years	Thoracic aorta	Yes		Deceased	2 weeks
Almeida et al (2011)^45^	60	M	Open	Polyester	Hemashield vascular graft (Boston Scientific Corporation)	Back pain; weight loss; fatigue; dyspnoea	CT/CT A; TTE	Malignancy	Percutaneous biopsy	9 years	Thoracic aorta	No	Chemotherapy; radiotherapy	Deceased	3 weeks
Bader et al (2016)^46^	72	M	Open	NS	NS	Knee pain	MRI/MRA; PET-FDG	Malignancy	Surgical explant	7 years	Popliteal	No	Surgery	Unknown	?
Chetouani et al (2017)^47^	81	M	Open	NS	Interposition bypass graft (not stated)	Inflammatory syndrome	CT/CT A; PET-FDG	NS	Surgical explant	1 year	Femoral	Yes	Surgery	Unknown	NS
Cherchi and Camparini (2017)^48^	64	F	Open	NS	Prosthetic femoropopliteal bypass graft (not stated)	Palpable mass; oedema	CT/CT A	NS	NS	NS	Popliteal	NS	NS	Deceased	17 days
Dietl et al (2018)^49^	59	M	Hybrid	NS	NS	Back pain; palpable mass	CT/CT A	Malignancy	Percutaneous biopsy	15 years	Thoracic aorta	Yes	Surgery	Deceased	within admission
Morimoto et al (2024)^50^	67	M	Open	Polyester	J-Graft Vascular Prosthesis (Japan Lifeline, Tokyo, Japan)	Asymptomatic	CT/CT A	Progressive aneurysmal disease	Surgical explant	7 years	Cardiac	Yes	Chemotherapy	Alive	12 months
This paper (1) 2024	75	M	Open	PTFE	Propaten Vascular Graft (8 mm, Ring-Reinforced ePTFE) (W. L. Gore & Associates, Flagstaff, AZ, USA)	Abdominal pain; back pain; nausea; vomiting; weight loss; syncope	CT/CT A; PET-FDG	Endoleak	Percutaneous biopsy of metastatic lesion	7 years	Femoral	Yes	Chemotherapy	Deceased	2 months

Abbreviations: CT, computed tomography; CT A, computed tomography angiography; MRA, magnetic resonance angiography; MRI, magnetic resonance imaging; NS, not stated; PET-FDG, positron-emission tomography–fluorodeoxyglucose; PTFE, polytetrafluoroethylene.

**Table 3.** Review of Literature: Angiosarcoma Following Open Procedure Without Prosthetic Material

	Age	Sex	Type of operation	Type of material	Prosthesis	Symptoms	Imaging	Differential diagnoses	Diagnosis made by	Time to tumour detection	Site of primary	Metastasis at time of diagnosis	Treatment	Outcome	Time to outcome
Nocturne et al (2010)^51^	81	M	Open	Vein	Saphenous vein	Lower limb claudication; palpable mass; weight loss; anorexia	CT/CT A; MRI/MRA; US	NS	Percutaneous biopsy	15 years	Popliteal	No	Radiotherapy	Deceased	3 months
Morris and Lee (2018)^52^	69	M	Open	Vein	Saphenous vein	Lower limb claudication; throbbing thigh pain; swelling	CT/CT A	Graft infection; pseudoaneurysm; malignancy	Surgical explant	2.5 years	Femoral	Yes	Surgery; radiotherapy	Deceased	3 months
Hartvigson et al (2019)^53^	72	M	Open	Vein	Saphenous vein	Chest pain	CT/CT A; Coronary angiography	Pseudoaneurysm	Surgical explant	18 years	Cardiac	Yes	Surgery; radiotherapy	Deceased	21 months with recurrence
Villaescusa Catalan et al (2019)^54^	74	M	Open	Vein	Saphenous vein	Leg pain; hypotension	CT/CT A; MRI/MRA	Pseudoaneurysm; malignancy; haematoma	Surgical explant	8 years	Femoral	No	Surgery	Deceased	2 months
Werra and Petzold (2021)^55^	83	M	Open	Vein	Saphenous vein	Palpable mass	CT/CT A	Endoleak	Surgical explant	3 years	Popliteal	Yes	Surgery	Deceased	1 months
Veterano et al (2022)^56^	58	M	Open	Vein	saphenous vein	Palpable mass	CT/CT A	ALI	Surgical explant	8 years	Femoral	Yes	Surgery	Deceased	2 months
Arts et al (2022)^57^	79	F	Open	Vein	Vein (not stated)	Lower limb claudication; palpable mass	CT/CT A; MRI/MRA; PET-FDG	CTLI	Surgical explant	9 years	NS	Yes	Surgery; chemotherapy	Deceased	8 months
May Lee et al (2022)^58^	84	M	Open	Vein	Saphenous vein	Palpable mass	NS	Malignancy	Surgical biopsy of metastatic lesion	3 years	Popliteal	No	Chemotherapy; radiotherapy	Alive	20 months

Abbreviations: CT, computed tomography; CT A, computed tomography angiography; MRA, magnetic resonance angiography; MRI, magnetic resonance imaging; NS: not stated; PET-FDG, positron-emission tomography—fluorodeoxyglucose; PTFE, polytetrafluoroethylene.

Presenting symptoms were typically non-specific, with most patients reporting localized pain at the prosthesis site, significant weight loss, general malaise, and fatigue. All cases where the symptomatology was described had presented with symptoms mimicking graft infection, or ongoing endoleak for EVAR cases. Elevated inflammatory markers and pain often led to initial misdiagnosis and delays in definitive treatment.

Computed tomography angiography was frequently the first line imaging modality and often identified structural anomalies around the graft, while magnetic resonance imaging (MRI) was less commonly performed due to artefacts from metallic components. Histopathological confirmation was most commonly achieved after surgical explantation, although percutaneous biopsy, biopsy of metastatic lesions, surgical thrombectomy specimens, and post-mortem examination also established the diagnosis in selected cases. Repeated biopsy attempts were occasionally necessary because of non-diagnostic samples, tissue fragmentation, or sampling artefact. Immunohistochemistry demonstrated strong positivity for vascular markers (CD31, factor VIII, and cytokeratins AE1/3).

Reported angiosarcomas were most commonly described in anatomical association with aortic or iliac reconstructions, although femoral and popliteal cases were also identified. Time from vascular procedure to tumour detection was inconsistently reported and should not be interpreted as true biological latency. Among cases with available data, the median time to tumour detection was 5.0 years after endovascular procedures, 8.0 years after open prosthetic or mixed/unclear procedures, and 8.0 years after open autologous-vein procedures. Given the small number of cases, heterogeneity of anatomical sites and procedures, incomplete reporting, publication bias, and uncertainty regarding the timing of tumour onset, no inferential comparison was performed. Material reporting was also incomplete: based on the material recorded in the included reports, 19 cases involved Dacron/polyethylene terephthalate, 11 involved PTFE alone, 8 involved autologous vein alone, 1 involved both vein and PTFE, and 10 had unspecified or unclear conduit/prosthesis material.

## DISCUSSION

Angiosarcoma is a rare and highly aggressive malignancy, originating from vascular, lymphatic, or endothelial cells. The variable symptomatology and imaging findings, and absence of molecular biomarkers, make this disease difficult to diagnose and treat. The clinical presentation depends on the growth pattern of the tumour, resulting in aneurysmal degeneration, intraluminal occlusion, or aortoiliac thromboembolic disease.^59^^,^^60^ Imaging findings were generally non-specific, with CT unable to adequately diagnose soft tissue malignancy. The FDG-PET frequently demonstrated avid disease but was non-specific, with uptake often interpreted as graft infection or inflammatory change. Given the non-specific clinical and imaging features of angiosarcoma, the gold standard for conclusive diagnosis remains adequate tissue sampling for histopathological examination and immunohistochemistry. Typical endothelial markers include CD31 (highest sensitivity and specificity), CD34, factor VIII–related antigen, and vascular endothelial growth factor (VEGF). The expression of cytokeratin may be helpful but also is present in poorly differentiated carcinomas.^2^^,^^4^^,^^61^

In the context of EVAR, the aortic aneurysmal sac is typically excluded from circulation by the covered endovascular stent graft, thereby making malignant growth within the sac difficult to identify. However, our third case report (patient 3) describes a case of endoluminal angiosarcoma with distal embolization, which is a presentation not identified among the other included reports. The precise site of tumour origin cannot be proven; however, the intraluminal distribution raises the possibility of origin from neointimal tissue within the endograft. There were also no type 1 or 3 endoleaks on surveillance to suggest that the angiosarcoma had initially arisen from within the aneurysm sac.

Experimental studies have suggested that malignant tumours can develop following the implantation of foreign bodies. In rodents, multiple synthetic polymers including Dacron and PTFE have been linked to sarcoma formation, though this has not been reproduced in humans.^14^^,^^15^ There have been other case reports demonstrating angiosarcoma and other sarcomas arising near other retained foreign bodies, including a retained bullet, a laparotomy sponge, and bone wax used for haemostasis at a bone graft donor site, with latency periods ranging from 4 months to 63 years.^14^^,^^62^ Based on rodent studies and case reports in humans, including the 3 reported in this series, it is thought that implanted foreign materials may potentially induce a wide range of sarcomas in humans, though a causal relationship has not been established.

The pathophysiological mechanisms underlying angiosarcoma development in this setting remain incompletely understood. It is uncertain whether tumourigenesis is primarily related to implanted foreign material, which is often inferred only from anatomical proximity and is not present in all reported cases, or whether it is mediated by post-procedural inflammation, oedema, and tissue remodelling. Notably, chronic lymphoedema, regardless of aetiology, is a recognized risk factor for angiosarcoma (Stewart-Treves syndrome) including cases associated with Milroy’s disease or chronic infections such as filariasis.^4^ This association raises the possibility that persistent oedema and chronic inflammation following vascular intervention may similarly promote malignant endothelial transformation even in the absence of prosthetic material.

The identification of angiosarcoma arising after autologous-vein bypass is therefore an important finding of this review, as it challenges a simplistic attribution of post-vascular-procedure angiosarcoma to synthetic graft material alone. Although vascular prostheses and endovascular devices may provide a substrate for chronic foreign-body reaction, local haemodynamic disturbance, neointimal injury, and persistent perivascular inflammation, comparable tumours have also been reported adjacent to autologous venous conduits where no synthetic material was implanted. These observations suggest that multiple, non-mutually exclusive mechanisms may contribute to tumour development, including chronic perivascular inflammation, repeated local tissue injury, altered flow dynamics, cumulative exposure to ionizing radiation from diagnostic or interventional surveillance, retained surgical material such as sutures or clips, and spontaneous tumourigenesis unrelated to the preceding vascular reconstruction.^7^^,^^8^

Cumulative radiation exposure may be a plausible but unproven contributor to angiosarcoma development following vascular procedures. Radiation-associated angiosarcoma is a recognized rare complication after therapeutic radiotherapy. For example, secondary breast angiosarcoma has been reported in approximately 0.04%-0.15% of patients treated with radiotherapy.^63^ In these cases, exposed tissues receive substantially higher, targeted absorbed doses than those typically encountered during diagnostic CT angiography or fluoroscopically guided abdominal aortic endovascular procedures, even when complex or repeated interventions are performed. Nevertheless, patients undergoing EVAR may accumulate radiation exposure over time through preoperative imaging, intraoperative fluoroscopy, postoperative CT surveillance, and adjunctive radiation-intensive reinterventions.^64^^,^^65^ In this review, many of the EVAR patients underwent additional complex endovascular procedures after the index vascular operation, although the radiation dose and timing were rarely reported. The predominance of EVAR-associated cases in this review is compatible with a possible role for cumulative radiation exposure; however, it may also reflect ascertainment and publication bias, greater imaging frequency, or the tendency for these tumours to mimic recognized EVAR complications such as endoleak or endograft infection. Therefore, although cumulative radiation exposure following vascular procedures remains a biologically plausible contributor to angiosarcoma development, this is only a potential hypothesis rather than an established aetiology or risk factor. Nonetheless, continued adherence to established radiation-minimization principles remains appropriate.

The interval between vascular procedure and tumour detection was prolonged, with a mean of 7.1 years and range of 5 months to 18 years among cases with available data. This interval highlights a significant diagnostic challenge, as early symptoms were frequently vague and non-specific, often mimicking graft infection, endoleak, or other post-procedural complications. However, it should not be interpreted as true biological latency, as the timing of tumour initiation cannot be determined from case reports.

However, it is important to note that when considering the number of vascular procedures performed each year, the development of angiosarcoma following a vascular procedure represents a very rare phenomenon. Unfortunately, as this literature consists entirely of case reports, it over-represents fatal and diagnostically dramatic presentations and omits the large denominator of uneventful grafts, so neither the EVAR predominance nor the fatal-outcome skew should be taken as representative of the underlying association. For context, our tertiary vascular centres each perform an average of 40 aortic procedures and 50 bypass procedures per year. To our knowledge, these are the only 3 cases of angiosarcoma following vascular procedures that have been identified across these centres over the past 15 years, demonstrating the rarity of this condition in the broader clinical landscape.

### Limitations

This systematic review is limited by the low quality of the available evidence, as all included studies were case reports or small case series. The absence of denominator data, control groups, cross-sectional studies, or systematic ascertainment of exposed populations precludes any reliable estimation of incidence, prevalence, attributable risk, or causality. In addition, the rarity and heterogeneity of angiosarcoma in this setting, together with inconsistent reporting and incomplete documentation across published cases, limits the ability to draw definitive conclusions regarding the relative contribution of vascular procedures, implanted materials, radiation exposure, chronic inflammation, or other potential risk factors.

Accordingly, the principal contribution of this review is not to establish a causal relationship, but to synthesize the reported clinical patterns of angiosarcoma across endovascular, open prosthetic, and autologous-vein reconstructions. The inclusion of autologous-vein cases is particularly important, as it challenges a purely prosthetic-material explanation and broadens the discussion to include multiple potentially overlapping mechanisms, including chronic perivascular inflammation, operative tissue injury, altered haemodynamics, cumulative ionizing radiation exposure, and sporadic tumourigenesis. The observed temporal and anatomical associations should therefore be interpreted as hypothesis-generating rather than causal.

Given the exceptional rarity of angiosarcoma after vascular procedures, the findings of this review do not support routine oncological surveillance after EVAR or bypass grafting. Rather, the clinical relevance lies in recognizing angiosarcoma as a rare but important differential diagnosis in atypical or refractory post-vascular-procedure presentations. Earlier consideration of tissue diagnosis may be warranted when presumed graft infection is not microbiologically confirmed, suspected endoleak is not demonstrated angiographically, perigraft or aneurysm-sac abnormalities progress despite intervention, systemic features such as weight loss are disproportionate, or repeated procedures fail to explain ongoing disease progression.

Given the extremely limited number of reported cases in the literature, further research, including through multicentre collaborative reporting and case aggregation, is needed to better characterize the aetiology, risk factors, clinicopathological features, and outcomes of this rare condition. Improved understanding of the mechanisms underlying delayed oncogenesis may ultimately inform risk stratification, diagnostic pathways, biomarker development, and earlier therapeutic intervention.

## CONCLUSION

This systematic review and case series synthesize the reported literature on angiosarcoma after vascular procedures across endovascular, open prosthetic and autologous-vein reconstructions. Its principal novel contribution is the description of apparent intraluminal angiosarcoma within an EVAR limb presenting with distal embolization, a clinical presentation not identified among the other included reports. The inclusion of autologous-vein cases challenges a purely prosthetic-material explanation and supports consideration of multiple overlapping mechanisms, including local tissue injury, chronic inflammation, altered haemodynamics, cumulative radiation exposure, and sporadic tumourigenesis. However, the available evidence consists of case reports and small case series only and, therefore, causality, incidence, and attributable risk cannot be established. Clinically, angiosarcoma should be considered as a rare differential diagnosis in atypical or refractory post-vascular-procedure presentations, particularly when presumed graft infection or endoleak is not microbiologically or angiographically confirmed, systemic symptoms are disproportionate, or repeated interventions fail to explain disease progression. Tissue diagnosis should be considered early in these patients.

## Supplementary Material

ivag236_Supplementary_Data

## Data Availability

The data underlying this article will be shared on reasonable request to the corresponding author.

## References

[1] Colas M , Gerazime A, Popescu D, et al Angiosarcoma: a population-based cancer registry descriptive study of 45 consecutive cases diagnosed between 1979 and 2016. Rare Tumors. 2020;12:2036361320979216. 10.1177/2036361320979216 PMC7739202 33403092

[2] Kordzadeh A , Askari A, Navi A, et al Primary angiosarcoma of aorta: a systematic review. Vascular. 2022;30:650-660. 10.1177/17085381211026491 34238080

[3] Rusthoven CG , Liu AK, Bui MM, et al Sarcomas of the aorta: a systematic review and pooled analysis of published reports. Ann Vasc Surg. 2014;28:515-525. 10.1016/j.avsg.2013.07.012 24485779

[4] Young RJ , Brown NJ, Reed MW, et al Angiosarcoma. Lancet Oncol. 2010;11:983-991. 10.1016/S1470-2045(10)70023-1 20537949

[5] Kumar V , Abbas AK, Aster JC, et al Robbins & Cotran Pathologic Basis of Disease. 10th ed. Elsevier; 2020.

[6] Buehler D , Rice SR, Moody JS, et al Angiosarcoma outcomes and prognostic factors: a 25-year single institution experience. Am J Clin Oncol. 2014;37:473-479. 10.1097/COC.0b013e31827e4e7b PMC3664266 23428947

[7] Cao J , Wang J, He C, et al Angiosarcoma: a review of diagnosis and current treatment. Am J Cancer Res. 2019;9:2303-2313. PMC6895451 31815036

[8] Penel N , Marreaud S, Robin YM, et al Angiosarcoma: state of the art and perspectives. Crit Rev Oncol Hematol. 2011;80:257-263. 10.1016/j.critrevonc.2010.10.007 21055965

[9] Radons J. The role of inflammation in sarcoma. Adv Exp Med Biol. 2014;816:259-313. 10.1007/978-3-0348-0837-8_11 24818727

[10] Keane Tahmaseb GC , Keane AM, Foppiani JA, et al An update on implant-associated malignancies and their biocompatibility. Int J Mol Sci. 2024;25:20240424. 10.3390/ijms25094653 PMC11083590 38731871

[11] Darawsha F , Kramer R, Raanani E, et al Angiosarcoma of the main pulmonary artery-hand-made conduit reconstruction. Interact CardioVasc Thorac Surg. 2022;35. 10.1093/icvts/ivac096 PMC9419682 35438178

[12] Fatima J , Duncan AA, Maleszewski JJ, et al Primary angiosarcoma of the aorta, great vessels, and the heart. J Vasc Surg. 2013;57:756-764. 10.1016/j.jvs.2012.09.023 23312835

[13] Nakata Y , Miyamoto K, Nishiyama K, et al Primary cardiac angiosarcoma with right atrial rupture. Interdiscip Cardiovasc Thorac Surg. 2025;40:20251001. 10.1093/icvts/ivaf227 PMC12836109 41032263

[14] International Agency for Research on Cancer. Surgical Implants and Other Foreign Bodies: IARC Monographs on the Evaluation of Carcinogenic Risks to Humans. World Health Organization; 1999.

[15] Oppenheimer BS , Oppenheimer ET, Stout AP, et al The latent period in carcinogenesis by plastics in rats and its relation to the presarcomatous stage. Cancer. 1958;11:204-213. 10.1002/1097-0142(195801/02)11:1<204::aid-cncr2820110132>3.0.co;2-8 13500316

[16] Almajan A , Parveen P, Saraceni C. Primary angiosarcoma of the aorta in association with an endurant stent graft. J Case Rep Images Oncology. 2021;7:1-6. 10.5348/100075z10aa2021cr

[17] Kano M , Nishisho T, Miyagi R, et al Intimal sarcoma arising from the common iliac artery presenting with artery occlusion: a case report and literature review. J Med Invest. 2019;66:205-208. 10.2152/jmi.66.205 31064943

[18] Schmehl J , Scharpf M, Brechtel K, et al Epithelioid angiosarcoma with metastatic disease after endovascular therapy of abdominal aortic aneurysm. Cardiovasc Intervent Radiol. 2012;35:190-193. 10.1007/s00270-011-0219-6 21735325

[19] Umscheid TW , Rouhani G, Morlang T, et al Hemangiosarcoma after endovascular aortic aneurysm repair. J Endovasc Ther. 2007;14:101-105. 10.1583/06-2004.1 17291154

[20] Stewart B , Manglik N, Zhao B, et al Aortic intimal sarcoma: report of two cases with immunohistochemical analysis for pathogenesis. Cardiovasc Pathol. 2013;22:351-356. 10.1016/j.carpath.2013.03.005 23628153

[21] Fenton J , Veenstra M, Bove P. Angiosarcoma involving native abdominal aortic aneurysm sac after endograft repair. Ann Vasc Surg. 2014;28: 490.e1-490.e4. 10.1016/j.avsg.2013.03.016 24200136

[22] Kimura S , Yonekura R, Umesue M. Angiosarcoma mimicking an infected pseudoaneurysm after graft replacement. Ann Thorac Surg. 2015;100:1114. 10.1016/j.athoracsur.2015.05.084 26354649

[23] Milite D , Pilon F, Ferrari A, et al Aortic epithelioid angiosarcoma after endovascular aneurysm repair. Ann Vasc Surg. 2016;35:207.e217-207.e221. 10.1016/j.avsg.2016.02.014 27238982

[24] Tiwari N , Krishna S, Ortega-Loayza AG. Aortic endograft and epithelioid sarcoma: a random association or causality? J Drugs Dermatol. 2016;15:897-899. 27391643

[25] Kamran M , Fowler KJ, Mellnick VM, et al Multimodality imaging approach towards primary aortic sarcomas arising after endovascular abdominal aortic aneurysm repair: case series report. Cardiovasc Intervent Radiol. 2016;39:940-947. 10.1007/s00270-015-1280-3 26721588

[26] de Almeida BL , Caria VP, Cavalcante SFA, et al [Not available]. J Vasc Bras. 2017;16:343-347. 10.1590/1677-5449.004117 PMC5944315 29930672

[27] Whittington EA , Duncan LD, McNally MM. Pleomorphic undifferentiated aortic sarcoma presenting as persistent endoleak after endovascular aneurysm repair. J Vasc Surg Cases Innov Tech. 2019;5:294-297. 10.1016/j.jvscit.2019.04.006 PMC6614707 31334403

[28] Yu PC , Aplin B, Reed AB. Angiosarcoma of the abdominal aorta after endovascular aneurysm repair. J Vasc Surg Cases Innov Tech. 2019;5:506-508. 10.1016/j.jvscit.2019.07.003 PMC6861570 31763509

[29] Presacco S , Agnes AL, Magalini S, et al Bleeding "sarcomatosis" as a rare presentation of vascular graft-related angiosarcoma: case report and review of the literature. BMC Surg. 2020;20:291. 10.1186/s12893-020-00966-7 PMC7678180 33218319

[30] Rota M , Arici V, Franciscone MM, et al Abdominal aorta angiosarcoma after endovascular aneurysm repair. Ann Vasc Surg. 2021;73:525-528. 10.1016/j.avsg.2020.12.011 33503501

[31] Derouane F , Brigitte H, Placide N. Epithelioid angiosarcoma arising after an endovascular aneurysm repair: case report and review of the literature. Acta Clin Belg. 2021;76:397-401. 10.1080/17843286.2020.1742489 32186994

[32] Takamura K , Kobayash H, Rubin BP, et al Aortic angiosarcoma in association with endovascular aneurysm repair: case report and review of the literature. Am J Case Rep. 2021;22:e931740. 10.12659/AJCR.931740 PMC8212843 34118148

[33] Walker K , Khan N, Scherer P. Angiosarcoma of the abdominal aorta after endovascular aneurysm repair : 18F-FDG PET/CT imaging findings. Clin Nucl Med. 2021;46:E200-E202. 10.1097/RLU.0000000000003363 33156052

[34] Pecceu S , Van Herzeele I, Deolet E, et al Angiosarcoma after endovascular aneurysm repair: case report and literature review. Acta Chir Belg. 2023;123:317-324. 10.1080/00015458.2021.2021718 34937527

[35] Cleret de Langavant B , Flaris AN, Dasnoy D. Epithelioid angiosarcoma after EVAR. A case report. Ann Vasc Surg. 2022;80:395.e391-395.e396. 10.1016/j.avsg.2021.10.053 34808264

[36] Azuma S , Shimada R, Maeda K, et al Residual aneurysmal angiosarcoma diagnosed after pulmonary multiple nodules appeared post additional embolization for type 2 endoleak. Ann Vasc Surg Brief Rep Innov. 2024;4:100323. 10.1016/j.avsurg.2024.100323

[37] Gegiia I , Savoie-White FH, Calabrino A, et al Ruptured aortic aneurysm with previous endovascular aneurysm repair in a patient with high-grade angiosarcoma. J Vasc Surg Cases Innov Tech. 2024;10:101610. 10.1016/j.jvscit.2024.101610 PMC11439839 39351206

[38] Fehrenbacher JW , Bowers W, Strate R, et al Angiosarcoma of the aorta associated with a Dacron graft. Ann Thorac Surg. 1981;32:297-301. 10.1016/s0003-4975(10)61056-9 6456705

[39] Weiss WM , Riles TS, Gouge TH, et al Angiosarcoma at the site of a Dacron vascular prosthesis: a case report and literature review. J Vasc Surg. 1991;14:87-91. 10.1016/0741-5214(91)90158-Q 1829490

[40] Brito DA , Carneiro F, Pedro M, et al Angiosarcoma associated with a vascular graft: case report. Int J Angiol. 1996;5:157-159. 10.1007/BF02043213

[41] Dargent JL , Vermylen P, Abramowicz D, et al Disseminated angiosarcoma presenting as a hemophagocytic syndrome in a renal allograft recipient. Transplant Int. 1997;10:61-64. 10.1111/j.1432-2277.1997.tb00538.x 9002154

[42] Kogon B , Kabeer M, Sawchuk AP, et al Angiosarcoma presenting as an occluded popliteal artery pseudoaneurysm. J Vasc Surg. 1998;27:970-973. 10.1016/s0741-5214(98)70280-0 9620152

[43] Ben-Izhak O , Vlodavsky E, Ofer A, et al Epithelioid angiosarcoma associated with a Dacron vascular graft. Am J Surg Pathol. 1999;23:1418-1422. 10.1097/00000478-199911000-00014 10555012

[44] Okada M , Takeuchi E, Mori Y, et al An autopsy case of angiosarcoma arising around a woven Dacron prosthesis after a Cabrol operation. J Thorac Cardiovasc Surg. 2004;127:1843-1845. 10.1016/j.jtcvs.2004.01.021 15173758

[45] Almeida NJ , Hoang P, Biddle P, et al Primary cardiac angiosarcoma: in a patient with a Dacron aortic prosthesis. Tex Heart Inst J. 2011;38:61-65. PMC3060733 21423471

[46] Bader T , Strobel K, Egger-Sigg M, et al FDG PET/MRI imaging of an angiosarcoma in a popliteal aneurysm and tibial head after popliteal graft. Clin Nucl Med. 2016;41:e426-427. 10.1097/RLU.0000000000001300 27405038

[47] Chetouani A , Perrin M, Drouet C, et al Aneurysmal angiosarcoma associated with vascular graft revealed by 18F-FDG-PET imaging. Radiol Case Rep. 2017;12:413-415. 10.1016/j.radcr.2016.12.005 PMC5417621 28491200

[48] Cherchi M , Camparini S. Late presentation of popliteal angiosarcoma after previously treated popliteal artery aneurysm. Eur J Vasc Endovasc Surg. 2017;54:463. 10.1016/j.ejvs.2017.07.015 28843982

[49] Dietl CA , Samedi VG, Langsfeld M. Malignant aortic tumors following open and endovascular implantation of prosthetic vascular grafts. Am J Cardiovasc Thorac Surg. 2018;3:1-7. 10.15226/2573-864X/3/3/00147

[50] Morimoto K , Miyasaka S, Ikeda Y, et al Primary angiosarcoma arising in the sinus of Valsalva: a case report. Int J Surg Case Rep. 2024;115:109308. 10.1016/j.ijscr.2024.109308 PMC10844813 38295745

[51] Nocturne G , Sellam J, Miquel A, et al Is sarcoma a complication of arterial femoro-popliteal bypass? Joint Bone Spine. 2010;77:358-360. 10.1016/j.jbspin.2010.03.018 20471894

[52] Morris R , Lee CJ. Development of angiosarcoma in a saphenous vein graft after femoral to above-knee popliteal artery bypass. Ann Vasc Surg. 2018;50:299.e9-299.e13. 10.1016/j.avsg.2018.01.087 29518506

[53] Hartvigson PE , Velamoor GR, Loggers ET, et al Cardiac angiosarcoma from a saphenous vein coronary artery bypass graft. Cureus. 2019;11:e5503. 10.7759/cureus.5503 PMC6816531 31667037

[54] Villaescusa Catalan JM , Martin IG, Cagigal Cobo ML. Popliteal angiosarcoma after bypass with autologous saphenous vein. Ann Vasc Surg. 2019;55:308.e301-308.e304. 10.1016/j.avsg.2018.06.034 30217704

[55] Werra UEM , Petzold PT. Epithelioid angiosarcoma of the popliteal artery - primary or secondary? A rare disease entity difficult to diagnose and easily overlooked-literature research and case report. Ann Vasc Surg. 2021;74:524.e521-524.e527. 10.1016/j.avsg.2021.02.010 33831522

[56] Veterano C , Teixeira S, Silva I, et al Angiosarcoma of a femoropopliteal bypass thrombosed vein graft. Vasc Endovascular Surg. 2022;56:448-453. 10.1177/15385744221077824 35258356

[57] Arts L , Roesti A, Haller C, et al Angiosarcoma after popliteal aneurysm exclusion surgery. A systematic review. Ann Vasc Surg. 2022;86:482-489. 10.1016/j.avsg.2022.06.012 35820530

[58] May Lee M , Pierobon E, Riva G, et al Angiosarcoma and vascular surgery: a case report and review of literature. Vasc Endovascular Surg. 2022;56:762-766. 10.1177/15385744221108248 35694959

[59] Squizzato F , Grego F. Primary aortic angiosarcoma presenting as progressive thrombosis of the thoracic aorta. Circ Cardiovasc Imaging. 2021;14:e010854. 10.1161/CIRCIMAGING.120.010854 33356416

[60] Park WK , Park KL, Cho YS, et al Intravascular epithelioid angiosarcoma in the abdominal aorta mimicking an infected aneurysm. Vasc Specialist Int. 2019;35:232-236. 20191231 10.5758/vsi.2019.35.4.232 PMC6941772 31915668

[61] Machado I , Giner F, Lavernia J, et al Angiosarcomas: histology, immunohistochemistry and molecular insights with implications for differential diagnosis. Histol Histopathol. 2021;36:3-18. 10.14670/HH-18-246 32885407

[62] Jennings TA , Peterson L, Axiotis CA, et al Angiosarcoma associated with foreign body material. A report of three cases. Cancer. 1988;62:2436-2444. 10.1002/1097-0142(19881201)62:11<2436::aid-cncr2820621132>3.0.co;2-j 3052791

[63] Torres KE , Ravi V, Kin K, et al Long-term outcomes in patients with radiation-associated angiosarcomas of the breast following surgery and radiotherapy for breast cancer. Ann Surg Oncol. 2013;20:1267-1274. 10.1245/s10434-012-2755-y PMC5036516 23224828

[64] Kalender G , Lisy M, Stock UA, et al Long-term radiation exposure in patients undergoing EVAR: reflecting clinical day-to-day practice to assess realistic radiation burden. Clin Hemorheol Microcirc. 2019;71:451-461. 10.3233/CH-170344 30248048

[65] Monastiriotis S , Comito M, Labropoulos N. Radiation exposure in endovascular repair of abdominal and thoracic aortic aneurysms. J Vasc Surg. 2015;62:753-761. 10.1016/j.jvs.2015.05.033 26169014

