# Supplementary material for: Angiosarcoma in the Setting of Vascular Procedures: A Systematic Review and Our Experiences
Source: Interdiscip Cardiovasc Thorac Surg. 2026 Aug 24;41(11):ivag236. doi: 10.1093/icvts/ivag236 (PMC13730550; doi:10.1093/icvts/ivag236)
Supplement: Supplementary file 1 [file ivag236_supplementary_data.docx]

Supplementary Appendix 1. Search strategy

The systematic literature search was performed from database inception to 31st October 2024. Searches were performed in PubMed/MEDLINE, Embase via Ovid, and the Cochrane Library. Searches were restricted to English-language studies involving human subjects. Conference abstracts, animal-only studies and non-English reports were excluded. Additional studies were identified by citation searching of included articles and relevant review articles.

| **Database** | **Search date** | **Search strategy** | **Records identified** |
| --- | --- | --- | --- |
| PubMed/MEDLINE | 31st October 2024 | (angiosarcoma OR hemangiosarcoma) AND (EVAR OR "endovascular aneurysm repair" OR "endovascular aortic repair" OR stent OR endovascular OR PTFE OR Dacron OR "bypass graft" OR "vascular prosthesis" OR "vascular prostheses") | 109 |
| Embase | 31st October 2024 | (‘EVAR’ OR ‘endovascular aneurysm repair’ OR ‘endovascular aortic repair’ OR stent OR endovascular OR PTFE OR ‘vascular graft’ OR Dacron OR bypass OR ‘vascular prosthesis’ OR ‘vascular prostheses’).mp. AND (angiosarcoma OR hemangiosarcoma).mp.  NOT (conference abstract status) | 251 |
| Cochrane Library | 31st October 2024 | (angiosarcoma OR hemangiosarcoma) AND (EVAR OR "endovascular aneurysm repair" OR "endovascular aortic repair" OR stent OR endovascular OR PTFE OR Dacron OR "bypass graft" OR "vascular prosthesis" OR "vascular prostheses") | 0 |
| Citation searching | 1st December 2024 | Reference lists of included articles and relevant reviews were manually searched for additional eligible case reports. | 5 |
